# Liraglutide and the management of overweight and obesity in people with schizophrenia, schizoaffective disorder and first-episode psychosis: protocol for a pilot trial

**DOI:** 10.1186/s13063-019-3689-5

**Published:** 2019-11-20

**Authors:** Clare Alexandra Whicher, Hermione Clare Price, Peter Phiri, Shanaya Rathod, Katharine Barnard-Kelly, Claire Reidy, Kerensa Thorne, Carolyn Asher, Robert Peveler, Joanne McCarthy, Richard Ian Gregory Holt

**Affiliations:** 1 Southern Health NHS Foundation Trust, Research and Development Department Tom Rudd Unit, Moorgreen Hospital, Botley Rd, West End, Southampton, SO30 3JB UK; 2 School of Health Sciences, Faculty of Environmental and Life Sciences, University of Southampton, Tremona Road, Southampton, SO16 6YD UK; 3 Barnard Health Research Limited, 42 Kilmiston Drive, Porchester, Fareham, PO16 8EG UK; 4 Liaison Psychiatry, College Keep, Terminus Terrace, Southampton, SO14 3DT UK; 5 Human Development and Health Academic Unit, Faculty of Medicine, University of Southampton, Tremona Road, Southampton, SO16 6YD UK

**Keywords:** Schizophrenia, Severe mental illness, Obesity, Overweight, Liraglutide, Feasibility, Pilot

## Abstract

**Background:**

People with severe mental illness (SMI) are two to three times more likely to be overweight and obese than the general population and this is associated with significant morbidity and premature mortality. Although lifestyle interventions can support people with SMI to lose weight, some are unable to make the necessary lifestyle changes or, despite making the changes, continue to gain weight.

**Objective:**

To assess the feasibility and acceptability of delivering a full-scale trial evaluating whether liraglutide 3.0 mg, a once-daily injectable therapy, may be an effective treatment of overweight and obesity in people with schizophrenia, schizoaffective disorder and first-episode psychosis.

**Methods:**

Design: a single-centre, double-blind, randomised, placebo-controlled trial.

Setting: mental health facilities within Southern Health NHS Trust.

Participants: 60 adults with schizophrenia, schizoaffective or first-episode psychosis prescribed antipsychotic medication will be recruited. Participants will be overweight or obese, defined by their baseline BMI which will be:

• BMI ≥ 30 kg/m^2^ or

• BMI ≥ 27 kg/m^2^ to < 30 kg/m^2^ in the presence of at least one weight-related consequence.

This is in concordance with the current EU licence for liraglutide (maximum dosage 3.0 mg).

Intervention: participants will be allocated in a 1:1 ratio using a computer-based randomisation programme to either once-daily subcutaneously administered liraglutide or placebo, titrated to 3.0 mg daily, for 6 months. All participants will receive standardised written information about healthy eating and exercise at their randomisation visit.

Outcomes: the main aim of the study is to gather data on recruitment, consent, retention and adherence. Qualitative interviews with a purposive sub-sample of participants and healthcare workers will provide data on intervention feasibility and acceptability. Secondary clinical outcome measurements will be assessed at 3 and 6 months and will include: weight, fasting plasma glucose, lipid profile, HbA_1c_ level; and the Brief Psychiatric Rating Scale.

**Discussion:**

This study should provide evidence of the potential benefits of liraglutide (maximum dosage 3.0 mg daily) on body weight and metabolic variables in people with schizophrenia, schizoaffective disorder and first-episode psychosis. It will also address the feasibility and acceptability of the use of liraglutide in mental health settings. This will inform the design of a longer outcome study that will be needed to determine whether any weight loss can be maintained in the long term.

**Trial registration:**

Universal Trial Number (UTN), ID: U1111-1203-0068. Registered on on 2/10/2017.

European Clinical Trials Database (EudraCT), ID: 2017-004064-35. Registered on 3/10/2017.

**Electronic supplementary material:**

The online version of this article (10.1186/s13063-019-3689-5) contains supplementary material, which is available to authorized users.

## Background

The prevalence of obesity in the general population has increased dramatically over the last 30 years and it seems likely that the environmental changes that have provoked these increases have also affected people with severe mental illness (SMI); in fact, the rates of overweight and obesity have increased even more rapidly in this cohort [1]. Obesity adversely affects the physical health and psychological well-being of people with SMI and if weight gain is attributed to treatment, this can lead to non-adherence and risk of relapse.

Schizophrenia is a major psychiatric disorder that alters the individual’s perception, thoughts, affect and behaviour and may involve a loss of insight and has a lifetime prevalence of approximately 1% [2]. Schizoaffective disorder is recognised as a separate condition to schizophrenia and is more likely to occur in women at a later age. This disorder affects an individual’s thoughts and emotions [3]. Although individuals with first-episode psychosis do not fulfil the diagnostic criteria for schizophrenia or schizoaffective disorder, 90–95% of people presenting with a non-affective psychotic episode (i.e. not mania and not depressive psychosis) will still meet the criteria for a schizophrenia spectrum disorder 2 years later. Mortality rates are increased two to three fold in people with SMI and life expectancy is reduced by 10–20 years. Approximately 75% of all deaths in people with schizophrenia are caused by physical illness with cardiovascular disease being the commonest cause [4]. Overweight and obesity contribute to this excess morbidity and mortality. Recent studies indicate that obesity is two to three times more common among people with SMI [5]. Obesity occurs early in the natural history of schizophrenia with a significant proportion of people with first-episode psychosis being overweight prior to any treatment. Substantial weight gain (> 7%) often occurs rapidly within 6–8 weeks after antipsychotic-treatment initiation [6]. While most weight gain occurs early in treatment, longer-term observational studies suggest that weight gain continues for at least 4 years albeit at a slower rate [7].

Individuals with schizophrenia are more likely to consume a diet that is rich in fat and refined carbohydrates while containing less fibre, fruit and vegetables than the general population [8]. Although there are fewer studies, people with first-episode psychosis also have poor diets [9]. Physical inactivity and the social and urban deprivation experienced by those with schizophrenia may contribute further to the increased obesity rates [8, 10]. There may be disease-specific effects of schizophrenia, such as genetic susceptibility, that have additive or synergistic actions to increase body weight further [5]. However, the most important factor related to weight gain in people with SMI is the use of antipsychotic medications, which are among the most obesogenic drugs. Weight gain is the commonest side effect of second-generation antipsychotic medication, affecting between 15 and 72% of patients [11]. Other psychotropic drugs are often prescribed to people with schizophrenia and include some antidepressants and mood-stabilising drugs, such as lithium and sodium valproate; these may also induce significant weight gain [12].

Both lifestyle and pharmacological interventions lead to significant reductions in body weight in the general population. Not only are the interventions clinically effective, they are also cost-effective because of the benefits of long-term improved health outcomes, including decreased mortality [13]. It is likely that similarly effective interventions for people with schizophrenia will also lead to improvements in health and would be a major step towards reducing the health inequalities experienced by people with schizophrenia. As the weight gain associated with antipsychotic medication result in some people discontinuing their medication, we hypothesis that effective weight-management strategies may also lead to improved adherence to antipsychotic medication and reduced relapse and hospitalisation rates.

Some studies have suggested that short-term lifestyle interventions could support weight reduction in people with SMI. A meta-analysis of non-pharmacological interventions in people with SMI [14] reported a mean reduction in weight of 3.12 kg over a period of 8–24 weeks. However, the results of longer-term studies are more mixed. A recent meta-analysis found significant weight loss in only two of six studies with interventions lasting longer than a year [15]. Most studies have included a mixed population of people with SMI and two large studies, which included only people with schizophrenia, found no effect of a lifestyle intervention on body weight [16, 17]. These latter studies suggest the weight management in people with schizophrenia may require a different approach from other SMIs such as bipolar disorder.

Given the challenges of implementing lifestyle change in people with schizophrenia and the lack of long-term effectiveness, alternative approaches are needed to manage overweight and obesity. A wide variety of treatments have been subject to clinical studies but currently no drug treatments are licensed for the treatment of antipsychotic-medication-associated weight gain or obesity in people with SMI with the exception of orlistat [18]. The long-term use of the latter, however, is extremely limited by high discontinuation rates, making it of little value in routine clinical practice [19].

To date, there have also been three completed trials of glucagon-like peptide 1 (GLP-1)-receptor agonists in people with SMI, two of which used exenatide and one used liraglutide (maximum dosage 1.8 mg) [20–22]. Liraglutide is a GLP-1-receptor agonist, with 97% homology to human GLP-1, which induces weight loss in humans mainly by reducing appetite and caloric intake, rather than increasing energy expenditure. There were contrasting results in the exenatide studies with one showing no difference between groups after 12 weeks of treatment [20] but in the other the exenatide arm had greater mean weight loss (− 5.29 vs − 1.12 kg; *P* = 0.015), and reduced glycosylated haemoglobin (HbA_1c_) levels (− 0.21% vs 0.03%; *P* = 0.004) [21]. In the liraglutide (maximum dosage 1.8 mg) study, glucose tolerance improved in the liraglutide group and body weight decreased compared with placebo (− 5.3 kg; 95% confidence interval (CI) − 7.0 to − 3.7 kg) [22].

Liraglutide is approved for the management of obesity at a dosage of 3.0 mg daily, which is higher than the dosage used to treat diabetes [23]. In a 56-week, double-blind trial involving 3731 participants without type-2 diabetes, 63.2% of the intervention arm compared with 27.1% in the placebo arm group lost at least 5% of their body weight, and 33.1% and 10.6%, respectively, lost more than 10% of their body weight [23]. We have, therefore, chosen to use liraglutide (maximum dosage 3.0 mg) as we postulate that a higher dosage of liraglutide may offer even greater weight loss in people with SMI than the 1.8-mg dosage employed in the previous study or other currently available GLP-1-receptor agonists.

### Aims and objectives

The aim of this pilot study is to undertake a double-blind, randomised controlled trial (RCT) of the use of liraglutide (maximum dosage 3.0 mg daily) in comparison to placebo in 60 obese or overweight people with schizophrenia, schizoaffective disorder or first-episode psychosis to assess the feasibility and acceptability of delivering a full-scale trial evaluating treatment with liraglutide in people with schizophrenia, schizoaffective disorder and first-episode psychosis.

## Methods

### Design

**Fig. 1:**
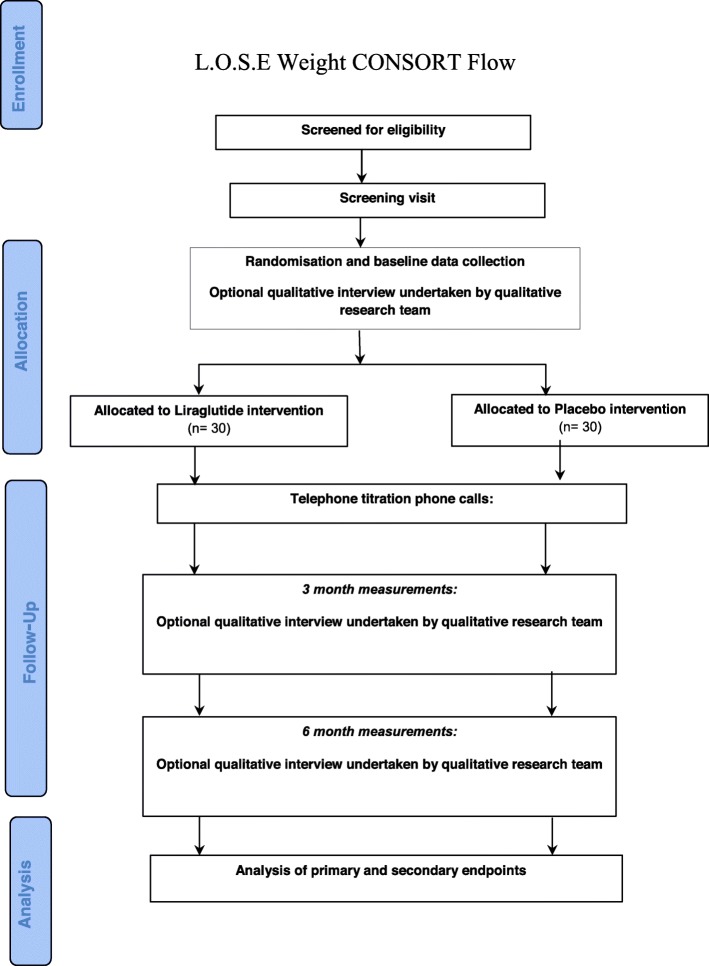
Consolidated Standards of Reporting Trials (CONSORT) study diagram

This study is a double-blind, randomised pilot study of the use of liraglutide (maximum dosage 3.0 mg daily) in comparison to placebo (Fig. 1). It is important to include a double-blind placebo for two main reasons; there is evidence that people would be less likely to consent to a trial that includes a placebo arm because of the risk of not receiving an active treatment. As our ability to recruit to the trial was one of our key aims, it is important to assess whether the inclusion of a placebo prevented us from recruiting to the trial. Previous experience from weight-management trials that include pharmaceuticals have been plagued by high dropout rates in the placebo arm as the participants are able to assess the effectiveness of treatment. To adequately power a full RCT of liraglutide, we will need to know the likely dropout rate in the placebo group in this patient population.

### Setting

The study will be take place in a variety of community and inpatient mental health locations in the Southern Health NHS Foundation Trust. Public and Patient Involvement was actively included in the development of the trial and will continue throughout the trial.

### Ethics approval and consent to participate

South Central – Hampshire B Research Ethics Committee (REC) approved the study on 17 April 2018 with REC reference: 18/SC/0085. Only those who agree to provide written informed consent will be included in the study. The study will be conducted in keeping with Good Clinical Practice (GCP) and the International Conference of Harmonisation (ICH) standards. The Trial Steering Committee (TSC) includes an independent chair and two other independent members, including a service user. The main trial investigators will also attend the TSC.

### Participants

Adults are eligible to participate in the study if they:

1. Are aged 18–75 years
2. Have a clinical diagnosis of schizophrenia or schizoaffective disorder (defined by *International Classification of Diseases, version 10* (*ICD-10*) codes F20 and F25) or first-episode psychosis using case note review. There is no limit on the duration of illness for those with schizophrenia or schizoaffective disorder but first-episode psychosis is defined as less than 3 years since presentation to the mental health team or the use of the first antipsychotic medication prescription
3. Are being treated with an antipsychotic medication, with a minimum duration of 1 month prior to entry in to the trial. No restriction is placed on the class or generation of the antipsychotic medication
4. Are able to give written informed consent
5. Ability and willingness to take liraglutide or placebo
6. Are able to speak and read English
7. Have a Body Mass Index (BMI, calculated as weight in kilograms divided by height in meters squared) *≥* 30 kg/m^2^ (obese), or *≥* 27 kg/m^2^ to < 30 kg/m^2^ (overweight) in the presence of at least one weight-related consequence such as dysglycaemia (pre-diabetes or type-2 diabetes), hypertension, dyslipidaemia or obstructive sleep apnoea

People are excluded from the study if they have a:

1. Physical illnesses, e.g. cancer, that could seriously reduce their life expectancy or ability to participate in the trial
2. A co-existing physical health problem that would, in the opinion of the principal investigator, independently impact on metabolic measures or weight, e.g. Cushing’s syndrome, poorly controlled type-2 diabetes defined by HbA_1c_ level > 8% (64 mmol/mol)
3. Inflammatory bowel disease and diabetic gastroparesis
4. Contraindications to liraglutide: hypersensitivity to liraglutide or to any of the excipients
  - Any condition which, in the investigator’s opinion, might jeopardise a participant’s safety or their compliance with the protocol
  - Family or personal history of multiple endocrine neoplasia type-2 or medullary thyroid carcinoma. Family is defined as a first-degree
  - relative
  - History or presence of pancreatitis (acute or chronic)
  - History of diabetic ketoacidosis
  - Any of the following: myocardial infarction, stroke, hospitalisation for unstable angina or transient ischaemic attack within the past 180 days prior to the day of screening
  - Participants presently classified as being in New York Heart Association Class IV
  - Planned coronary, carotid or peripheral artery revascularisation known on the day of screening
  - Renal impairment measured as an estimated glomerular filtration rate (eGFR) value of < 30 ml/min/1.73 m^2^ as defined by the Kidney Disease Improving Global Outcomes (KDIGO) 2012 classification
  - Impaired liver function, defined as alanine aminotransferase (ALT) ≥ 2.5 times the upper normal limit at screening
  - Proliferative retinopathy or maculopathy requiring acute treatment
  - Presence or history of malignant neoplasms within the past 5 years prior to the day of screening. Basal and squamous-cell skin cancer and any carcinoma in situ is allowed
5. Use of other pharmacological products for weight management
6. Mental illnesses that could seriously reduce their ability to participate in the trial, including significant suicidality
7. Current pregnancy or a desire to become pregnant. Mothers who are less than 6 months post-partum or breastfeeding will also be excluded. In line with the current EU licence and advice from the Medicines and Healthcare products Regulatory Agency (MHRA), sponsor and manufacturer, any women who may become pregnant during the trial but are unwilling to use a highly effective method of birth control (e.g. such as implants, injectables, combined oral contraceptives, intrauterine devices, sexual abstinence or having a vasectomised partner) will not be eligible for the trial
8. Significant alcohol or substance misuse which, in the opinion of the principal investigator, would limit a participant’s ability to participate in the trial
9. A diagnosis or tentative diagnosis of psychotic depression or mania
10. A primary diagnosis of learning disability or cognitive impairment which would impair a participant’s ability to self-administer trial medication
11. Lack of capacity. Those who lose capacity any time during the study will not be eligible to continue and will be withdrawn from the study immediately with no further study procedures carried out
12. History of type-1 diabetes.
13. Current or previous use of incretin-based therapies (GLP-1-receptor agonist or dipeptidyl peptidase 4 (DPP-4) inhibitors) or insulin

### Sample size

This pilot trial will explore the feasibility and practical issues of conducting a future definitive trial and estimate the important parameters to help its design. In this regard, sample size is based on the need to estimate study parameters within a reasonable degree of precision rather than on hypothesis testing. Simulation work by Sim et al. (2012) recommended a minimum of 50 participants (25 per group) in order to achieve the pilot/feasibility objectives [24]. A further paper by Whitehead et al. recommended pilot trial sample sizes per treatment arm of 75, 25, 15 and 10 for standardised effect sizes that are extra small (≤ 0.1), small (0.2), medium (0.5) or large (0.8), respectively [25]. In the previous liraglutide study in people with SMI, 10% of the liraglutide arm and 2% in the placebo arm had dropped out of the trial after 16 weeks [22]. Although dropout from obesity trials is non-linear as those on active treatment drop out earlier because of side effects while those on placebo drop out later because of lack of efficacy, we assume a conservative dropout rate at 6 months of between 15 and 20%. As such, we will need to recruit at least 60 participants (30 per group) to provide robust estimates that will inform the design of the definitive trial.

In a pilot trial looking at the use of liraglutide (maximum dosage 1.8 mg) of 214 potential participants assessed for eligibility, 103 were randomised. Of the 111 excluded, 86 did not meet the final inclusion/exclusion criteria, 23 declined to participate and 2 had too severe a degree of mental illness to participate [22]. However, in a similar study examining the use of once-daily exenatide in people with schizophrenia, out of 123 potentially eligible participants, only 28 were randomised with 63 declining to participate [21]. We used these data to estimate our screen-to-randomisation rate.

### Selection

The study will be promoted within clinical teams and in areas where community mental health services are delivered in the Southern Health NHS Foundation Trust. It is estimated that ~ 70% of those with a recorded diagnosis of schizophrenia or one of its subtypes attending community and inpatient settings of the Southern Health NHS Foundation Trust fulfil the inclusion criteria. This equates to approximately 500 individuals across the trust. Assuming that 30% of patients were willing to take part, we would be able to recruit up to 60 patients for the study within 12 months. Confirming these screening and enrolment rates is part of the reason for performing the study.

In addition, the Early Intervention in Psychosis team in Southampton receives approximately 20 new referrals a month. Although these individuals do not fulfil the diagnostic criteria for schizophrenia, we intend to include them in the study for the following reasons:

- Up to 80% of individuals treated with antipsychotic medication during a first episode of psychosis gain more than 7% of their body weight within 12 weeks of treatment.
- People with first-episode psychosis are more likely to develop weight-induced metabolic abnormalities and consequently the benefits may be greater

Participants will be advised verbally and in writing that they will be able to end their participation in the study at any point without affecting their clinical care. The investigators will also have the right to withdraw participants from the study if they lose capacity, develop any of the exclusion criteria or miss their 3-month study visit. The rationale for the withdrawal will be recorded and dated in the Case Report Form (CRF) accordingly. Participants withdrawing from the trial treatment will be encouraged to undergo the same final clinical evaluations.

### Randomisation

After baseline assessments, participants will be randomised to either daily subcutaneously administered liraglutide (maximum dosage 3.0 mg) or matching placebo. Equal numbers of participants will be randomised to each arm of the trial using simple randomisation with permuted, blinded, block size. Novo Nordisk prepares and provides the subject randomisation list (SRL) using a computer-based programme. All participants, carers and study personnel except the pharmacy team will be blinded to treatment assignment. This includes the statistician undertaking the data analysis.

Emergency un-blinding will be undertaken if a participant develops an adverse event that requires knowledge of the treatment, an overdose of trial medication or there is a clinical need to start a participant on medication which has a risk of interaction with the trial drug.

### Intervention

Before screening, all potential participants will be provided with written information about the trial including the most common adverse event and the procedures involved in the study. All participants will receive standardised written information about healthy eating, physical activity, alcohol and smoking.

Liraglutide will be used according to the current EU licence for Saxenda®; the starting dose will be 0.6 mg per day. Participants will be taught face-to-face about using the injection pen and will be witnessed giving the first injection. Participants will be given an instruction leaflet to take away with them. The dose will be increased each week by 0.6 mg to a maximum dosage of 3.0 mg per day. Participants who do not tolerate up-titration will remain on the highest tolerable dosage. Each participant will attend 4-weekly visits where concomitant medications and adverse events will be documented. In addition at the baseline, 3- and 6-month visits they will also have clinical data collected (secondary endpoints) including drawing blood samples. The blood samples will be analysed for fasting plasma glucose (FPG), lipid profile and HbA_1c_ level_._ Participants will be invited at baseline and study completion to take part in one-to-one interviews with a psychologist, trained and experienced in qualitative research methodology, to explore expectations and experience of their participation in the trial.

### Outcome measures

#### Primary objective

The primary objective of the pilot trial is to investigate the feasibility and acceptability of delivering a full-scale trial evaluating whether liraglutide 3.0 mg, once-daily injectable therapy, may be an effective treatment of overweight and obesity in people with schizophrenia, schizoaffective disorder and first-episode psychosis.

In order to achieve our primary objective, the study will gather data on:

- Time to reach the recruitment target
- The number of eligible participants required to be screened in order to reach the recruitment target. Key characteristics and reasons for not joining the trial will be recorded, in line with the Consolidated Standards of Reporting Trials (CONSORT) criteria for clinical trials
- To estimate participant attrition rate
- To estimate adherence to the investigational medicinal product (IMP)

#### Secondary exploratory outcomes

To estimate effect size and standard deviation (SD) of the change in weight at 6 months in order to inform a power calculations for a fully powered RCT based on this feasibility pilot study.

Changes in waist circumference, BMI, FPG, HbA_1c_ level, blood pressure, lipid profile, adverse events and Brief Psychiatric Rating Scale (BPRS) score at 3 and 6 months will also be assessed.. The BPRS is an instrument used for assessing the positive, negative and affective symptoms of psychotic disorders, especially schizophrenia. The BPRS will, therefore, be used to assess any changes in mental health during the trial.

Follow-up windows for 3- and 6-month follow-ups will be defined as minus and plus 2 weeks to allow for missed appointments.

**Fig. 2:**
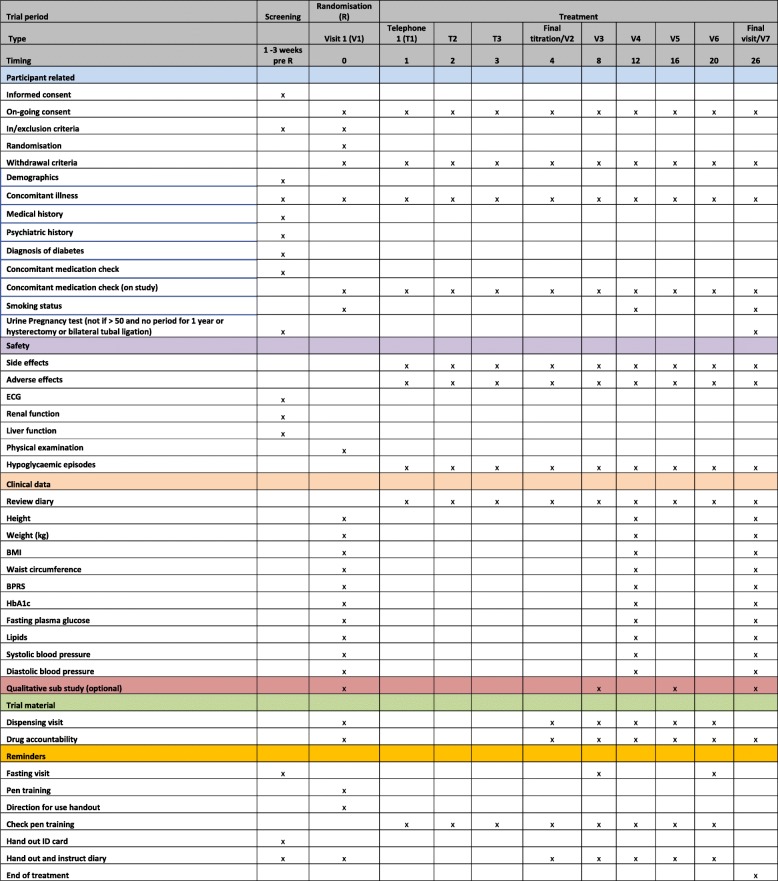
Schedule of outcome measures and trial-related activities

A schedule of follow-up activities is shown in Fig. 2.

### Safety assessments

Heart rate and glycaemia assessments will be taken at baseline and at 3 and 6 months post randomisation. Adverse events (AEs) are defined as the side effects listed in the Summary of Product Characteristics (SmPC) and include nausea, vomiting and diarrhoea. Medication error and laboratory outliers will also be considered as AEs. AEs will be monitored every 4 weeks.

Serious Adverse Events (SAEs) are defined as per GCP. Any SAE which a member of the study team deems to be associated with the trial intervention will be assessed by the principal investigator.

There are SAEs that are expected for the patient population:

- Psychiatric hospitalisation
- Worsening of psychiatric symptoms
- Self-harm
- Suicide attempt
- Death from suicide

If the investigator deems that any of these expected events are at least possibly related to the study drug then these will be reported as serious adverse reactions (and not events).

All SAEs that are both ‘unexpected’ (that is, the type of event is not listed in the protocol as an expected occurrence); and ‘related’ (that is, it resulted from administration of any of the research procedures) will be reported to the sponsor for expedited reporting to the TSC and the REC. No serious adverse outcomes are anticipated associated with use (or not) of the trial medication; therefore, no interim statistical analysis is planned regarding safety; however, the TSC will review all SAEs periodically.

### Data analysis

Data will be analysed in accordance with the trial’s detailed Statistical Analysis Plan (SAP) (Additional files 1 and 2), a summary of the main methods are as follows:

All data will be analysed based on the intention-to-treat population. Continuous variables will be analysed by either mean or median, with groups compared statistically using either a paired *t* test or a Mann-Witney *U* test as appropriate. Categorical variables will be presented as *n* (%) with groups compared using chi-squared or Fisher’s exact tests. The difference in weight change between groups will be analysed using generalised linear models (GLMs), both unadjusted and adjusted for covariates that are identified as potential confounders in univariate testing. All statistical tests will be two-sided with statistical significance assumed at 0.05.

Key characteristics and reasons for not joining the trial will be recorded for all participants screened. A CONSORT diagram will be used to summarise this information and reasons will also be provided if applicable.

Demographics and person-reported outcome data at baseline will be summarised using number and proportion, mean and SD or median and interquartile range (IQR) as appropriate.

#### Analysis of primary objectives

This study focusses on the feasibility of recruiting from this patient population for a fully powered RCT. The following will be reported:

- Time to reach recruitment target (in weeks)
  - ◦ Mean number of participants recruited per week
  - ◦ Rate of successful screens
  - ◦ Participant attrition rate
- Adherence to the IMP (defined as the proportion of medication used by each person ranging from 0 to 100%)
  - ◦ Either mean (SD) or median (IQR) adherence will be presented as appropriate
  - ◦ Number of participants using at least 70% of the prescribed trial medication. Adherence to the IMP is defined as the number of empty cartridges returned at each visit divided by the total number of cartridges prescribed

#### Analysis of secondary exploratory outcomes

Changes in weight (defined as weight in kilograms at 3 or 6 months minus weight in kilograms at randomisation), BMI, waist circumference, BPRS score, HbA1c level, FPG, lipid level, systolic and diastolic blood pressure, and adherence to randomised treatment (including the effect of the using the optional text-messaging reminder service or not), type of diabetes medication, change in type or dose of diabetes medication, type of antipsychotic medication, change in type or dose of antipsychotic medication between the two treatment groups will be summarised and tested for significance, as will the number of participants experiencing a weight loss of at least 5% from baseline to 3 to 6 months.

Change in body weight between the two groups at 26 weeks will be further analysed using a GLM adjusted for baseline weight and other covariates.

#### Missing data

Analysis will be completed using list-wise deletion of missing data.

Participants with and without missing data will be compared for differences in demographic and physiological data where possible.

#### Harms

The number (and percentage) of participants experiencing each AE/SAE will be presented for each treatment arm categorised by severity.

### Qualitative component of study

One-to-one semi-structured interviews will be held with a sub-sample of liraglutide-treated participants and healthcare professionals delivering the intervention to provide data on the drug treatment. Purposive sampling will ensure diversity in terms of demographic and disease characteristics. Participants (10–12 from each trial arm) will be interviewed until data saturation is met.

Semi-structured interview-topic guides will contain questions intended to elicit themes outlined in the existing published literature, after exploring more general open questions on the experience and acceptability of the treatments. Interviews will be held before, during and at the end of the trial.

For mental health workers, key themes relate to the perception of pharmacological interventions as part of the care-coordinating role, workload, the need for specialist knowledge and views on client adherence. We will use May’s normalisation process model as a theoretical framework to understand the conditions necessary to support the introduction, embedding and integration of a weight intervention as a routine element of care. All semi-structured interviews will be audio-taped and fully transcribed. Content and thematic analysis will use the National Centre for Social Research ‘Framework’ approach.

## Trial status

Recruitment opened on 2 July 2018 and we aim to complete recruitment by 31 July 2019. Protocol version 1.6, dated 22 May 2018, is being used.

## Discussion

Obesity adversely affects the physical health and psychological well-being of people with SMI. If weight gain is attributed to treatment, this can lead to non-adherence and risk of relapse. The provision of an effective intervention to reduce the burden of overweight and obesity in people with schizophrenia, schizoaffective disorder and first-episode psychosis would improve physical health and reduce the risk of developing obesity-related illnesses as well as improving psychological well-being.

This pilot study is a double-blind, randomised, placebo-controlled trial investigating the use of once-daily liraglutide subcutaneous injection in obese or overweight people with schizophrenia, schizoaffective disorder or first-episode psychosis. It aims to explore the feasibility and practical issues of conducting a future definitive RCT evaluating weight change with liraglutide in overweight or obese people with SMI. This feasibility trial should estimate important parameters to help its design. One potential limitation of this feasibility trial is the funding by the investigational drug manufacturer. In order to mitigate against this bias, the trial was sponsored by the Southern Health NHS Foundation Trust, which has the responsibility for the initiation, management, conduct, analysis, reporting and publication of the trial. Although Novo Nordisk is providing support financially and the product for the trial, Novo Nordisk is not involved in the conduct, management and delivery of the trial. Additionally, the initial idea, rationale and design for the trial came from the chief investigator. Nevertheless, the results of this study will need to be confirmed in a fully powered, investigator-led trial.

This research should contribute to the development of effective weight-management intervention programmes for people with SMI. It should provide information on whether injectable GLP-1-receptor agonists are an acceptable weight-loss medication in this group of people. Further, potentially more effective, GLP-1-based medications are in development including once weekly and orally administered versions which may prove to be a better option for people with SMI if once daily injections are shown to be feasible. .

## Additional files

Additional file 1: Statistical Analysis Plan (SAP). L. O. S. E. Weight Pilot Study. (DOCX 39 kb)

Additional file 2: Standard Protocol Items: Recommendations for Interventional Trials (SPIRIT) 2013 Checklist: recommended items to address in a clinical trial protocol and related documents. (DOCX 121 kb)

## Data Availability

Not applicable.

## Abbreviations

AE: Adverse event
BMI: Body Mass Index
BPRS: Brief Psychiatric Rating Scale
CI: Confidence interval
CRF: Case Report Form
eGFR: Estimated glomerular filtration rate
FPG: Fasting plasma glucose
GCP: Good Clinical Practice
GLM: Generalised linear model
GLP-1: Glucagon-like peptide 1
ICH: International Conference on Harmonisation
RCT: Randomised controlled trial
REC: Research Ethics Council
SAE: Serious adverse event
SD: Standard deviation
SmPC: Summary of Product Characteristics
TSC: Trail Steering Committee
