# Supplementary material for: Liraglutide and the management of overweight and obesity in people with schizophrenia, schizoaffective disorder and first-episode psychosis: protocol for a pilot trial
Source: Trials. 2019 Nov 20;20:633. doi: 10.1186/s13063-019-3689-5 (PMC6868690; doi:10.1186/s13063-019-3689-5)
Supplement: Supplementary file 1 — Statistical Analysis Plan (SAP). L. O. S. E. Weight Pilot Study. (DOCX 39 kb) [file 13063_2019_3689_MOESM1_ESM.docx]

**Statistical Analysis Plan (SAP)**

**L. O. S. E. Weight Pilot Study**

**Authors**: Dr Clare Whicher, Ms Kerensa Thorne

(Southern Health NHS Foundation Trust)

**Version**: 3.0

**Date**: 31/05/2019

**This document has been written based on information contained**

**in the study protocol version 1.6, dated 22^nd^ May 2018**.

**1 INTRODUCTION**

L. O. S. E. Weight is a double blind randomised pilot study investigating the use of once daily liraglutide subcutaneous injection in comparison to placebo in obese or overweight people with schizophrenia, schizoaffective disorder or first episode psychosis. It aims to explore the feasibility and practical issues of conducting a future definitive randomised controlled trial (RCT) evaluating weight change with liraglutide in overweight or obese people with severe mental illness. This feasibility trial will estimate important parameters to help its design.

**2 OBJECTIVES**

**2.1 Primary Objective**

The primary objective of the trial is to gather data on feasibility for a fully powered trial, as follows:

1. Time to reach recruitment target.
2. The number of eligible participants required to be screened in order to reach recruitment target. Key characteristics and reasons for not joining the trial will be recorded, in line with the CONSORT criteria for clinical trials.
3. To estimate participant attrition rate.
4. To estimate adherence to the investigational medicinal product.

**2.2 Secondary exploratory outcomes**

To estimate effect size and standard deviation (SD) of the change in weight at 26 weeks in order to inform a power calculation for a fully powered RCT based on this feasibility pilot study.

The secondary objective is to test the null hypothesis that there is no difference in weight loss between treatment groups. Changes in waist circumference, body mass index, fasting plasma glucose, HbA1c, blood pressure, lipid profile at 12 and 26 weeks will also be assessed for statistical significance.

**3 TRIAL METHODS**

**3.1 Trial design**

The trial is a single centre, double blind, randomised, placebo-controlled trial. Treatment allocation is a 1:1 ratio. Participants are randomised to either liraglutide or matched placebo control.

**3.2 Randomisation**

Each randomisation is via simple randomisation with permuted block size. The randomisation process is described in full within the clinical trial protocol.

**3.3 Sample Size**

This study is a pilot trial aiming to explore feasibility, practical issues of conducting a future definitive trial and estimate important parameters to help its design. In this regard, sample size is based on the need to estimate study parameters within a reasonable degree of precision rather than on hypothesis testing. Simulation work by Sim et al (2012) recommended a minimum of 50 participants (25 per group) in order to achieve pilot/feasibility objectives.[1] Assuming a dropout rate at 6 months of between 15% to 20%, we will need to recruit at least 60 participants (30 per group) to provide robust estimates that will inform the design of the definitive trial.

In a pilot trial looking at the use of liraglutide (maximum dose 1.8 mg) of 214 potential participants assessed for eligibility 103 were randomised. Of the 111 excluded 86 actually did not meet final inclusion/exclusion criteria, 23 declined to participate and 2 had too severe degree of mental illness to participate. [2] However, in a similar study, the use od exenatide in people with schizophrenia, out of 123 potentially eligible participants only 28 were randomised with 63 declining to participate. [3] We used these data to estimate our screened to randomised rate (see table below).

**3.4 Interim analysis**

This study focusses on the feasibility of recruiting from this patient population for a fully powered RCT. The recruitment rate will be reviewed at 12 weeks post study start (i.e. 24/9/18) compared to the planned trigger points in the table below and the recruitment strategy revised if necessary. No serious adverse outcomes are anticipated associated with use (or not) of the trial medication; therefore no interim statistical analysis is planned regarding safety, however the trial steering committee will be reviewing all SAEs periodically.

| **Trigger point** | **Screened** | **Randomised (per month)** | **Randomised (cumulative)** | **Date due** |
| --- | --- | --- | --- | --- |
| 1 | 5 (5) | 2 | 2 | End July 2018 |
| 2 | 8 (13) | 4 | 6 | End August 2018 |
| 3 | 8 (21) | 4 | 10 | End Sept 2018 |
| 4 | 10 (31) | 4 | 14 | End Oct 2018 |
| 5 | 10 (41) | 5 | 19 | End Nov 2018 |
| 6 | 12 (53) | 3 | 22 | End Dec 2018 |
| 7 | 12 (65) | 5 | 27 | End Jan 2019 |
| 8 | 12 (77) | 6 | 33 | End Feb 2019 |
| 9 | 12 (89) | 6 | 39 | End March 2019 |
| 10 | 12 (101) | 6 | 45 | End April 2019 |
| 11 | 12 (113) | 6 | 51 | End May 2019 |
| 12 | 10 (123) | 5 | 56 | End June 2019 |
| 13 | 0 (123) | 4 | 60 | End July 2019 |

**3.5 Data quality control**

Data will be recorded via paper CRFs and entered by a dedicated member of staff onto an electronic data management system which applies appropriate range and format checks on entry.

**4 STATISTICAL PRINCIPLES**

**4.1 Statistical significance**

All applicable statistical tests will be 2-sided and will be performed using a 5% significance level. All confidence intervals presented will be 95% and two-sided.

**4.2 Analysis populations**

The data will be analysed based on the intention-to-treat population; all randomised participants, regardless of their eligibility, according to the treatment they were randomised to receive will be included. Due to the feasibility nature of this study a per-protocol analysis will not be necessary.

**4.3 Screening data and participant flow**

Key characteristics and reasons for not joining the trial will be recorded for all participants screened. A CONSORT diagram will be used to summarise the number of participants who were:

- Estimated number of eligible participants in the recruitment area (from a Current Research Information System (CRIS) search of the Southern Health database)
- Pre-screened for eligibility via medical notes
- Invited for screening visit; accepted and not accepted*
- assessed for eligibility at screening visit; eligible and not eligible*
- eligible and randomised
- eligible but not randomised*
- received the randomised allocation
- did not receive the randomised allocation*
- lost to follow-up*
- discontinued the intervention*
- Randomised and included in the primary analyses

*reasons will be provided.

**4.4 Baseline participant characteristics**

Participants will be described with respect to age, gender, ethnicity, smoking status, diagnosis of type 2 diabetes (yes/no), time since diagnosis of diabetes if applicable, diabetes treatment if applicable, type of psychiatric diagnosis and time since this diagnosis, type of antipsychotic medication, weight, BMI, waist circumference, brief psychiatric rating scale (BPRS), HbA1c, fasting plasma glucose (FPG), lipids, systolic and diastolic blood pressure at baseline, both overall and separately for the two randomised groups.

Categorical baseline data will be summarised by numbers and percentages. Continuous baseline data will be summarised by mean and SD if data are normal or median and IQR if data are skewed. Tests of statistical significance will not be undertaken for baseline characteristics; rather the clinical importance of any imbalance will be noted.

**5 ANALYSIS**

**5.1 Outcome definitions**

1. Time to reach recruitment target is defined as the time from first participant screened to randomisation of the 60^th^ participant.
2. Number of participants required to be screened in order to reach recruitment target is defined as the number of participants attending a screening visit.
3. Participant attrition rate is defined as the number of participants not available for follow-up at the final study visit as per the research protocol.
4. Adherence to the investigational medicinal product is defined as the number of empty cartridges returned at each visit by trial participants divided by the total number of cartridges prescribed. Adherence will be analysed both as a continuous variable and by the number of participants using at least 70% of prescribed trial medication over 12 weeks and 26 weeks.

**5.2 Analysis methods**

**5.2a Analysis of primary objectives**

1. Time to reach recruitment target will be reported as a number (in weeks). The mean number of participants recruited per week will also be presented with 95% confidence interval
2. Number of participants required to be screened: the rate of successful screens will be evaluated as the number of participants randomised divided by the number of participants screened; presented as proportion with 95% CI.

The following will be analysed at 12 and 26 weeks, both overall and within treatment group:

1. Participant attrition rate: will be evaluated as the number of participants not available for follow-up, divided by the number of participants randomised; presented as proportion with 95% CI.

- Adherence to the investigational medicinal product – (defined as the proportion of medication used by each person ranging 0-100%)
  1. Either mean (sd) or median (IQR) adherence will be presented as appropriate
  2. Number of participants using at least 70% of prescribed trial medication with 95% CI

**5.2b Analysis of secondary exploratory outcomes**

Changes in weight (defined as weight in kilograms (kg) at 3 or 6 months minus weight in kg at randomisation), BMI, waist circumference, brief psychiatric rating scale (BPRS), HbA1c, fasting plasma glucose (FPG), lipids, systolic and diastolic blood pressure, and adherence to randomised treatment (including the effect of the using the optional text messaging reminder service or not), type of diabetes medication, change in type or dose of diabetes medication, type of antipsychotic medication, change in type or dose of antipsychotic medication between the two treatment groups will be reported using mean (SD) or median (IQR) according to the distributions, and compared statistically using either paired t-test or Mann-Witney U test. The number of participants experiencing a weight loss of at least 5% from baseline to 12 weeks and 26 weeks will also be reported and tested for significance.

We will then use a generalised linear model (GLM) adjusted for baseline in order to compare the change in body weight between the two groups at 26 weeks. This will be done

1. Unadjusted for covariates
2. Adjusted for any covariates that are significantly different between the two treatment groups in the univariate analysis described above

**5.3 Missing data**

Analysis will be completed using listwise deletion of missing data.

Participants with and without missing data will be compared for differences in demographic and physiological data where possible, by looking at appropriate summary statistics with statistical tests, as follows:

- Mean (sd) with t-test or Median (IQR) with Mann-Whitney test for continuous data
- N (%) with either chi-squared test or Fisher’s Exact test for categorical data

Differences between the participants will be taken into account in deducing the feasibility of a full study.

**5.4 Harms**

The number (and percentage) of patients experiencing each AE/SAE will be presented for each treatment arm categorised by severity. For each patient, only the maximum severity experienced of each type of AE will be displayed. The number (and percentage) of occurrences of each AE/SAE will also be presented for each treatment arm. No formal statistical testing will be undertaken.

**5.5 Statistical software**

The analysis will be carried out using IBM SPSS Statistics 19. Other packages such as R may be used if necessary.

**6 REFERENCES**

1. Sim, J., *The size of a pilot study for a clinical trial should be calculated in relation to considerations of precision and efficacy.* J Clin Endocrinol, 2012. **65**(3): p. 301-308.

2. Larsen, J.R., et al., *Effect of Liraglutide Treatment on Prediabetes and Overweight or Obesity in Clozapine- or Olanzapine-Treated Patients With Schizophrenia Spectrum Disorder: A Randomized Clinical Trial.* JAMA Psychiatry, 2017. **74**(7): p. 719-728.

3. Siskind, D.J., et al., *Treatment of clozapine-associated obesity and diabetes with exenatide in adults with schizophrenia: A randomized controlled trial (CODEX).* Diabetes Obes Metab, 2017.
